# Flavonoids: Antioxidants Against Atherosclerosis

**DOI:** 10.3390/nu2080889

**Published:** 2010-08-12

**Authors:** Davide Grassi, Giovambattista Desideri, Claudio Ferri

**Affiliations:** 1 Department of Internal Medicine and Public Health, University of L’Aquila, L’Aquila, 67100 Italy; Email: giovambattista.desideri@cc.univaq.it (G.D.); claudio.ferri@cc.univaq.it (C.F.); 2 University of L’Aquila Department of Internal Medicine and Public Health Viale S. Salvatore, Delta 6 Medicina, 67100 Coppito, L’Aquila, Italy

**Keywords:** oxidative stress, endothelial dysfunction, atherosclerosis, antioxidants, flavonoids

## Abstract

Oxidative stress results from an imbalance between excessive formation of reactive oxygen species (ROS) and/or reactive nitrogen species and limited antioxidant defences. Endothelium and nitric oxide (NO) are key regulators of vascular health. NO bioavailability is modulated by ROS that degrade NO, uncouple NO synthase, and inhibit synthesis. Cardiovascular risk conditions contribute to oxidative stress, causing an imbalance between NO and ROS, with a relative decrease in NO bioavailability. Dietary flavonoids represent a range of polyphenolic compounds naturally occurring in plant foods. Flavonoids are potentially involved in cardiovascular prevention mainly by decreasing oxidative stress and increasing NO bioavailability.

## 1. Introduction

Oxidative stress has been considered a mechanism involved in the pathogenesis of ischemic heart disease and atherogenesis, in cancer and other chronic diseases, and it also plays a major role in the aging process [1,2,3]. Oxidative damage by free radicals has been well investigated within the context of oxidant/antioxidant balance. Indeed, oxidative stress describes various deleterious processes resulting from an imbalance between the excessive formation of reactive oxygen and/or nitrogen species and limited antioxidant defences. In this regard, cardiovascular risk factors significantly cause oxidative stress, which contributes to a disruption in the balance between nitric oxide (NO) and reactive oxygen species, with a resulting relative decrease in NO bioavailability. The resulting endothelial dysfunction has been supposed to be the first step of atherosclerosis. Further, the majority of cardiovascular diseases follow from complications of atherosclerosis. In addition, an important initiating event for atherosclerosis may well be the transport of oxidized low-density lipoprotein across the endothelium into the artery wall [1,2,3]. Diet and nutrition play a fundamental role in cardiovascular prevention and in maintaining physiological homeostasis. Recent literature emphasizes the potential therapeutic effects of micronutrients found in natural products, indicating positive applications for controlling the pathogenesis of chronic cardiovascular disease driven by cardiovascular risk factors and oxidative stress [1,4,5]. Nutritional compounds that display anti-inflammatory and antioxidant effects have specific applications in preventing oxidative stress-related injury, which characterizes the pathogenesis of cardiovascular disease. Polyphenolic compounds, mainly flavonoids, are ubiquitous dietary components. Dietary flavonoids represent a diverse range of polyphenolic compounds that occur naturally in plant foods. Flavonoids from food have been reported to be potentially involved in cardiovascular prevention mainly by decreasing oxidative stress and increasing NO bioavailability. They are able to modulate genes associated with metabolism, stress defence, drug metabolizing enzymes, detoxification and transporter proteins [1,4,5]. Their overall effect is protective in overcoming damaging effects of cardiovascular risk factors and in delaying the onset of atherosclerosis [1,4,5,6]. Thus, they have naturally been associated with the hypothesis that their redox activities may confer them with specific health benefits. Their prevalence in plant derived foods has supported this point of view and inspired new research for human intervention trials with flavonoid-rich food items in order to investigate their ability to protect from cardiovascular risk [1,4,5]. In recent years, there has been a remarkable interest in scientific studies focusing on oxidative stress. The reasons seem to be the knowledge about reactive oxygen and nitrogen species metabolism, the definition and clinical role of markers for oxidative damage, the evidence linking cardiovascular diseases and oxidative stress, and the identification of flavonoids and other dietary polyphenol antioxidants able to act as bioactive molecules with health benefits deriving from flavonoid-rich diet. In this brief review, the potential role for flavonoids in prevention of oxidative stress by modulating endothelial mechanisms responsible for the atherosclerotic process will be discussed.

## 2. Oxidative Stress and Endothelial Dysfunction

All aerobes including plants, aerobic bacteria, and humans, suffer damage when exposed to oxygen concentrations higher than normal, signifying that they have no excess of antioxidant defenses [7]. Halliwell and Gutteridge [7] defined free radicals as molecules or molecular fragments containing one or more unpaired electrons in atomic or molecular orbitals. This unpaired electron(s) usually gives a considerable degree of reactivity to the free radical. Radicals derived from oxygen represent the most important class of radical species generated in living systems [1,2,3,6,7,8,9,3,6]. Reactive oxygen species are produced by various oxidase enzymes, including nicotinamide-adenine dinucleotide phosphate (NADPH) oxidase, xanthine oxidase, uncoupled endothelial NO synthase (eNOS), cyclooxygenase, glucose oxidase, lipooxygenase, and mitochondrial electron transport [6,7,8,9]. An imbalance between oxidants and antioxidants in favor of the oxidants, potentially leading to damage, has been defined “oxidative stress” [9]. The term describes a metabolic condition of cells, organs, or the entire organism characterized by an oxidative overload [7,8,9]. Indeed, the excess reactive oxygen species can damage cellular lipids, proteins, or DNA impairing their normal function. Because of this, oxidative stress has been implicated in a number of human diseases as well as in the ageing process. The delicate balance between beneficial and harmful effects of free radicals is a very important aspect of living organisms [7,8,9]. Superoxide anion is considered the “primary” reactive oxygen species and can further interact with other molecules to generate “secondary” reactive oxygen species, either directly or prevalently through enzyme- or metal-catalysed processes [8]. Further, superoxide anion may react with other radicals including NO. The product peroxynitrite is also a very powerful oxidant and belongs to the reactive nitrogen species, *i.e.* derived from NO. The sources of reactive oxygen species are a variety of cell types, including vascular smooth muscle cells, endothelial cells (ECs) and mononuclear cells. Several lines of evidence demonstrate that oxidative stress plays an important role in the pathogenesis and development of cardiovascular diseases. The susceptibility of vascular cells to oxidative stress is a function of the overall balance between the degree of oxidative stress and the antioxidant defence capability. Further, NADH/NADPH oxidase is the most important source of reactive oxygen species in the vasculature and inactivation of NADH/NADPH oxidase may contribute to the improvement in endothelial dysfunction in patients with atherosclerosis [10,11]. Increased production of reactive oxygen species has been demonstrated to impair endothelial function in humans [11,12,13]. An imbalance of reduced production of NO and increased production of reactive oxygen species may be involved in impaired endothelium-dependent vasodilation in patients with cardiovascular risk factors and diseases. It has been reported that a vicious cycle of endothelial dysfunction and oxidative stress leads to development of atherosclerosis [1,2,3,4,5,6,7,8,9,10,11]. NO is constitutively generated in endothelial cells from its precursor L-arginine by the action of eNOS (converting L-arginine to citrulline) in the presence of cofactors such as tetrahydrobiopterin. NO diffuses to the vascular smooth muscle cells and activates the soluble guanylate cyclase, which leads to cGMP-mediated vasodilatation. The cGMP acts as a second messenger leading to many of the biological effects of NO such as relaxation of smooth muscle and inhibition of platelet aggregation [12,13,14]. Shear stress is a key activator of eNOS in normal physiology, and this adapts organ perfusion to changes in cardiac output [12,13,14]. NOS appears in at least three isoforms: NOS 1 (or cNOS), a constitutive isoform in central and peripheral nervous system as well as in platelets; NOS 2 (or iNOS), the inducible form of the enzyme, is found in myocytes, macrophages and ECs and is activated by immunological and inflammatory stimuli; NOS 3 (or eNOS), an endothelial isoform which plays a very important role in the vascular homeostasis. The constitutive eNOS-derived NO production is beneficial for the cardiovascular system, while the large amounts of NO produced by iNOS as part of inflammatory processes favors the synthesis of the cytotoxic peroxynitrite, promoting atherogenesis [13,14].

In normal vascular physiology, NO plays a key role to maintain the vascular wall in a quiescent state by inhibiting inflammation, cellular proliferation, and thrombosis [4,5,10,11,12,13,14]. The endothelium maintains vascular homeostasis through multiple complex interactions with cells in the vessel wall and lumen. Further, a “healthy” endothelium maintains vascular tone and structure by regulating the balance between vasodilation and vasoconstriction [11,12,13]. On the contrary, in pathologic conditions, particularly with the presence of cardiovascular risk factors, the endothelium undergoes functional and structural alterations, thus losing its protective role and becoming a proatherosclerotic structure [11,12,13]. In the earliest stages, the principal endothelial alteration is merely functional and addressed as “endothelial dysfunction.” The fundamental feature of this condition is the impaired NO bioavailability. This can be the consequence of either a lowered production by eNOS or, more frequently, of an increased breakdown by reactive oxygen species [4,5,11,12,13,14]. The fundamental change involved in this process is a switch in signalling from a NO-mediated silencing of cellular processes toward activation by redox signalling. Further, it is intriguing to note that eNOS, which normally helps maintain the quiescent state of the endothelium, can switch to generate reactive oxygen species in certain circumstances as part of endothelial dysfunction. In this case, interestingly, eNOS itself can paradoxically produce superoxide, a process referred to as "eNOS uncoupling." Reduced levels of tetrahydrobiopterin or L-arginine lead to uncoupling of reduced NADPH oxidation and NO synthesis, with oxygen as terminal electron acceptor instead of L-arginine, resulting in the generation of superoxide by eNOS [1,2,3,4,5,11,12,13,14,15]. Degradation of tetrahydrobiopterin by reactive oxygen species is associated with an additional downregulation of eNOS [11,12,13,14,15]. According to this, it has been showed that supplementation of tetrahydrobiopterin improves endothelial function *in vitro* and in clinical studies involving patients with cardiovascular risk factors. Moreover, it has been observed that the grade of oxidative stress correlated with a deficiency of tetrahydrobiopterin, thus suggesting tetrahydrobiopterin deficiency and decreased eNOS activity may cause endothelial dysfunction in atherosclerotic patients through an increase in oxidative stress [10,11,12,13,14,15]. Thus, the ability of eNOS to regulate both the quiescent and the altered endothelial phenotype puts this enzyme at the center of endothelial homeostasis [10,11,12,13,14,15]. Following the above evidence we could affirm that intact function and integrity of the endothelium play a pivotal role for cardiovascular health. Insults to ECs by cardiovascular risk factors reduce or abolish the NO functions. In patients at high cardiovascular risk, the decline in endothelial NO bioavailability is attributed to: (1) the reduced expression of eNOS; (2) the deficiency of substrate or cofactors for eNOS and a deficient activation of eNOS caused by impaired cellular signaling; (3) the decreased capacity of ECs to synthesize and/or release NO; or (4) the inactivation of synthesized NO by reactive oxygen species [11,12,13,14]. All these abnormalities might induce endothelial dysfunction that is considered the earliest step in the pathogenesis of atherosclerosis. Both traditional and novel cardiovascular risk factors initiate a chronic inflammatory process that is accompanied by a loss of vasodilator and antithrombotic factors and an increase in vasoconstrictor and prothrombotic products [11,12,13,14]. It has been hypothesised that this endothelial-dependent vascular imbalance is critical, not only in the initiation and progression of atherosclerosis, but also in the transition from a stable to an unstable disease state with the precipitation of acute vascular events [11,12,13,14]. It has been supposed that a dysfunctional endothelium may promote plaque activation, which leads to a higher plaque vulnerability, thus, successively inducing plaque destabilization and rupture. Concordant with this, the magnitude of endothelial dysfunction is an important and independent predictor of future development of cardiovascular risk and events [16,17,18]. Endothelial dysfunction is also relevant to the later stages of the disease and seems to play a role in acute coronary syndromes [16]. In patients with established atherosclerosis, disturbed vasomotion associated with endothelial activation may contribute to transient myocardial ischemia and angina pectoris [17,18]. Abnormalities of endothelial function are also present in patients with traditional atherosclerotic risk factors, including hypercholesterolemia, tobacco smoking, diabetes, and hypertension, prior to any clinical manifestations of atherosclerosis [17,18,19,20]. Endothelial dysfunction has been shown to be associated with an increase in reactive oxygen species in atherosclerotic animal models and human subjects with atherosclerosis [17,18,19,20]. Several investigators have shown that impaired endothelium-dependent vasodilation is found in the forearm, coronary, and renal vasculature in patients with cardiovascular disease [17,18,19,20]. Endothelial dysfunction also has prognostic implications and is associated with an increased risk of future cardiovascular events [21,22,23]. Particularly, a study [23] evaluating endothelium-dependent (plethysmography of forearm blood flow in response to acetylcholine) and -independent vasodilation (sodium nitroprusside), reported that endothelial dysfunction and increased vascular oxidative stress predicted the risk of cardiovascular events in patients with coronary artery disease. Patients experiencing cardiovascular events had lower vasodilator responses to acetylcholine (*p *< 0.001), but greater benefit from vitamin C administration (*p *< 0.01). Thus, these findings confirm the concept that oxidative stress may contribute not only to endothelial dysfunction but also to coronary artery disease activity [23]. Further, nutritional, or dietary oxidative stress denotes a disturbance of the redox state resulting from excess oxidative load or from inadequate nutrient supply favouring prooxidant reactions. Postprandial oxidative stress has been described in postprandial hyperglycemia and/or hyperlipidemia and is associated with a higher risk for atherosclerosis, diabetes, and obesity [9]. Additionally, endothelial function is impaired in the postprandial state of hyperlipidemic and hyperglycaemic subjects. Thus, postprandial oxidative stress could be considered as an important factor modulating cardiovascular risk. In spite of this, flow-mediated dilation (FMD) was not affected by administration of a low-fat meal or a high-fat meal that included 1 g vitamin C and 800 IU vitamin E [24]. Therefore, it could be suggested that postprandial oxidative stress might be attenuated when dietary antioxidants are supplied together with a meal rich in oxidized or oxidizable lipids. Ingestion of dietary polyphenols, e.g., from wine, cocoa, or tea, improves endothelial dysfunction and lowers the susceptibility of LDL lipids to oxidation [9,24].

In the clinical setting, it seems of relevance to select appropriate interventions for both endothelial function and oxidative stress. This might be expected to greatly improve clinical outcomes. Therefore, given these findings one could also argue that the therapeutic correction of endothelial dysfunction may cause an improvement of prognosis in patients with cardiovascular risk factors or cardiovascular disease. 

## 3. Dietary Strategies Against Oxidative Stress

Based on evidence of the importance of oxidative stress in cardiovascular damage, there has been great interest in developing strategies that target reactive oxygen species in the treatment of cardiovascular diseases. Therapeutic approaches that have been considered include mechanisms to increase antioxidant bioavailability or to reduce reactive oxygen species generation. The mechanisms involved in radical scavenging activity are complex, determined by the structure of the compound, redox status of the environment and interactions with other agents. In this regard, it is of interest that purified micronutrients isolated from natural products may be less effective than a combination observed in the natural product due to synergistic effects of interacting agents.

Relatively scant data still characterize the *in vivo* implications of these findings. Nevertheless, there have been studies suggesting that the regular or occasional consumption of polyphenols and/or flavonoid-rich foods exerts beneficial effects on blood pressure, insulin resistance, endothelial function, and oxidative stress [4,5,6]. Dietary antioxidants constitute a large group of compounds that differ in mechanism of action, bioavailability and side effects. A systematic analysis of the role of the various antioxidants in chronic diseases is retarded by the difficulty of employing death or clinical events as end points in intervention studies. Therefore, valid markers for oxidative stress, which show dose response and are sensitive to changes in dietary supply of antioxidants, are potentially of great value when trying to establish healthy dietary patterns, or when one component, like cocoa, tea or red wine, is studied [4,5,6,24,25,26,27]. Epidemiologic studies indicate that diets rich in fruit and vegetables are associated with a decreased incidence of adverse cardiovascular events, such as coronary artery disease and stroke [24,25,26,27]. This effect was ascribed, at least in part, to the high content of antioxidants, in particular polyphenolic compounds, such as flavonoids, in plant-based foods [4,5,6,24,25,26,27]. In this context, cocoa, some chocolates, red wine, and tea received much attention, because they are particularly rich in flavonoids, phytochemicals with strong antioxidant properties *in vitro* [4,5,6]. Several lines of evidence suggest that flavonoids, a major class of polyphenols, are important bioactive constituents of the above mentioned foods and that there may be a causal relationship between flavonoid consumption and improvements in cardiovascular function. 

## 4. Polyphenols: Structure and Classification

**Figure 1:**
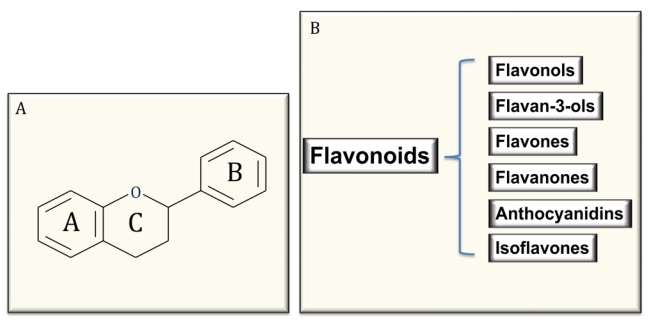
Common structure of flavonoids (A) and classification (B).

Polyphenols are the most abundant antioxidants in our diet and are common constituents of foods of plant origin and are widespread constituents of fruits, vegetables, cereals, olive, dry legumes, chocolate and beverages, such as tea, coffee and wine [4,5,6,28,29]. Despite their wide distribution, the health effects of dietary polyphenols have been attentively studied by nutritionists only in recent years [28,29]. Polyphenols comprise a wide variety of molecules that have a polyphenol structure (*i.e.*,several hydroxyl groups on aromatic rings), but also molecules with one phenol ring, such as phenolic acids and phenolic alcohols. Polyphenols are divided into several classes, according to the number of phenol rings that they contain and to the structural elements that bind these rings to one another. Flavonoids comprise the most common group of plant polyphenols. More than 8,000 different flavonoids have been described and since they are prerogative of the kingdom of plants, they are part of our diet with a daily total intake amounting to 1 g/day, which is higher than all other classes of phytochemicals and known dietary antioxidants. In fact, the daily ingestion of β-carotene, vitamin C, and vitamin E from food often is estimated < 100 mg/day intake [1,4,26,27,28,29]. Flavonoids are structured as a common carbon skeleton of diphenyl propanes, two benzene rings (ring A and B) joined by a linear three-carbon chain (C6-C3-C6) usually forming an oxygenated heterocycle nucleus, the flavan nucleus (ring C). Depending on the structural complexity of flavonoids, particularly on the oxidation state of the central ring C, flavonoids are themselves subclassified as flavonols, flavones, flavanones, flavanols or flavan-3-ols (catechins and their oligomers: proanthocyanidins), isoflavones, and anthocyanins [4,27,28,29] (Figure 1). Regarding their distribution in foods, flavonoids could be considered ubiquitous antioxidants, nevertheless, some of them could be specific to particular foods, while others, such as quercetin, are found in all plant products [27,28,29]. Furthermore, a number of different factors, such as harvesting, environmental factors, and storage, may affect the polyphenol content of plants. Additional variability in flavonoid content could be expected in finished food products because its availability is largely dependent on the cultivar type, geographical origin, agricultural practices, post-harvest handling and processing of the flavonoid containing ingredients [27,28,29].

## 5. Flavonoids, Oxidative Stress and Endothelial Effects

**Figure 2:**
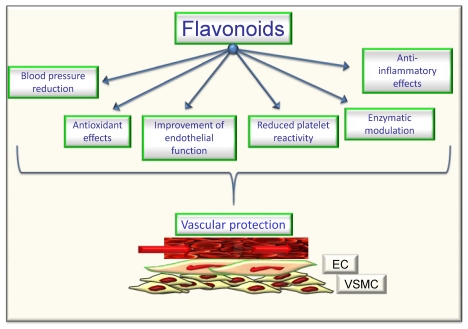
Potential effects of flavonoids on cardiovascular protection.

The review of epidemiological and mechanistic studies supports the role of flavonoids, particularly cocoa and tea flavanols, in protecting the cardiovascular system [4,5,6,26,27]. In this regard, many studies have shown that flavonoids demonstrate protective effects against the initiation and progression of atherosclerosis [1,4,5,6]. The bioactivity of flavonoids and related polyphenols appears to be mediated through a variety of mechanisms, though particular attention has been focused on their direct and indirect antioxidant actions. As antioxidants, polyphenols may protect cell constituents against oxidative damage and, therefore, limit the risk of various degenerative diseases associated to oxidative stress. In particular, it has been shown that the consumption of polyphenols limits the development of atheromatous lesions, inhibiting the oxidation of low density lipoprotein [30], which is considered a key mechanism in the endothelial lesions occurring in atherosclerosis. Moreover, mechanisms of antioxidant effects may include: (1) suppressing reactive oxygen species formation either by inhibition of enzymes or chelating trace elements involved in free radical production; (2) scavenging reactive oxygen species; and (3) upregulating or protecting antioxidant defences [1,4,5,6]. Further, they can also satisfy most of the antioxidant criteria. It has been hypothesized that their antioxidant properties may protect tissues against oxygen free radicals and lipid peroxidation [31,32]. Most flavonoids are effective radical scavengers. This property by itself does not imply a beneficial effect, because after scavenging a flavonoid radical is formed. A very reactive flavonoid radical would propagate rather than interrupt the deleterious events initiated by the radical attack. However, a flavonoid radical with high stability will not readily react. As a consequence, this flavonoid will act as an antioxidant [4,31,32,33]. Furthermore, prooxidant effects of polyphenols have been described [34], having opposite effects on basic cell physiological processes: as antioxidants they may improve cell survival, as pro-oxidants they might induce apoptosis and block cell proliferation, thus acting as anti-carcinogenic factor [35]. However, emerging findings suggest a variety of potential mechanisms of action of polyphenols in preventing disease, which may be independent of their conventional antioxidant activities. Increasing evidence suggests that flavonoids might exert several other specific biological effects such as the inhibition or reduction of different enzymes and the interaction with signal transduction pathways and cell receptors [1,33,36]. In keeping to this, it was reported that flavonoids such as epigallocatechin gallate and resveratrol were able to inhibit the transcription factors NFkB and AP-1 via interaction with upstream signalling pathways (IKK phosphorylation, MAPK phosphorylation and PI3K/Akt phosphorylation) and/or by decreasing pro-inflammatory mediators (TNF-a, IL, PGE2) and activities of proinflammatory enzymes (COX 2, iNOS) [1,28]. Concordantly, a recent study by Rezzani *et al.* [37] showed that treatment with antioxidants such as pycnogenol and melatonin may protect the vasculature in an experimental model of genetic hypertension (spontaneously hypertensive rats). Of interest, they observed that both melatonin and pycnogenol treatment increased MMP2 expression toward that observed in control rats. Further, melatonin or pycnogenol administration decreased iNOS protein expression compared with untreated spontaneously hypertensive rats. A higher COX-2 expression was observed in untreated spontaneously hypertensive rats compared with controls, which was prevented by both melatonin and pycnogenol administration. Of note, although the endothelial response to acetylcholine was not fully normalized in treated spontaneously hypertensive rats, a significant improvement in mesenteric small resistance artery endothelial function was observed in treated rats compared with controls. Total aortic collagen content was significantly greater in untreated (corresponding to type I collagen-fibrotic collagen) compared with control rats, and with spontaneously hypertensive rats treated with melatonin or pycnogenol (prevalence of type III collagen–constitutive collagen) [6,37]. The molecular mechanisms by which phytochemicals interact with signal transmission cascades are not precisely known, and it has been hypothesised that the downregulation of transcription factors may be due to the direct scavenging of reactive oxygen species [1,4,5,6]. It has been observed [29] that exposure of human ECs to (–)-epicatechin (the most important flavanol in cocoa) resulted in elevation of cellular levels of NO and cyclic GMP and in protection against oxidative stress elicited by pro-inflammatory agonists. Therefore, the authors supposed that endothelial NO metabolism rather than general antioxidant activity is a major target of dietary flavanols and that NADPH oxidase activity may represent a crucial site of action. NO protection against oxidants and increased NO bioavailability have been suggested to be primarily responsible for the vascular health benefits derived from flavonoid-rich food and beverage consumption, particularly in conditions that are known to be characterized by either increased oxidant production or decreased antioxidant defense mechanisms or both [1,4,5,6,31,32,33,36,38]. It has been reported [39] that a single dose of a flavanol-rich cocoa drink transiently increased NO bioactivity in human plasma and significantly reversed endothelial dysfunction in patients with at least a cardiovascular risk factor. Further, authors observed a correlation between NO-dependent FMD and levels of nitrosylated and nitrosated species. This suggested that flavan-3-ols induce arterial dilation via their effects on NO bioavailability [39]. Of interest, a recent study by Davison *et al.* [40], investigated the effects of cocoa flavanols and regular exercise in overweight and obese adults. They showed that, compared to low-flavanol, high-flavanol cocoa acutely increased FMD by 2.4% (P < 0.01) and chronically (over 12 weeks; P < 0.01) by 1.6%. Further, flavanol-rich cocoa intake reduced insulin resistance by 0.31% (P < 0.05), diastolic blood pressure by 1.6 mmHg and mean arterial blood pressure by 1.2 mm Hg (P < 0.05). Expanding on this, we demonstrated that flavanol-rich dark chocolate administration significantly increased the endothelium-dependent FMD of the brachial artery also positively affecting additional cardiovascular risk factors in healthy subjects [41] as well as in hypertensive patients with and without glucose intolerance [41,42]. Further, we also showed that epigallocatechin-3-gallate and (–)-epicatechin induced a dose-dependent NO-mediated vasorelaxation in isolated rat aortic rings precontracted by phenylephrine [5]. In addition, with regard to tea flavanols, an early study by Duffy *et al.* [43] showed that short- as well as long-term black tea consumption reversed endothelial vasomotor dysfunction in patients with coronary artery disease. Accordingly, our research group [44] reported that black tea (containing increasing doses of flavonoids but very similar of caffeine) ingestion dose-dependently improved endothelial function. Black tea increased FMD from 7.8% (control) to 9.0, 9.1, 9.6 and 10.3% after the different flavonoid doses, respectively (*p *= 0.0001). Of interest, even 100 mg/day (less than 1 cup of tea) increased FMD compared with control (*p *= 0.0113). Furthermore, FMD after 800 mg/day was significantly higher than control (*p *< 0.0001) but also higher than 100 mg/day (*p *= 0.0121) and 200 mg/day (*p *= 0.0275) administration [44]. All data support the significant putative role of chocolate and tea flavanols in protecting the cardiovascular system, by ameliorating endothelial function and likely by decreasing oxidative stress. Indeed, in addition to directly scavenging NO, flavonoids can act to rapidly down-regulate peroxynitrite generation from NO. The ability of flavonoids and flavonoid-rich foods and beverages to reduce NO oxidation and increase NO bioavailability appears to contribute significantly to its vascular benefits [1,4,5,6] and thus, finally, to protect against atherosclerosis [1,2,3,4]. Moreover, the polyphenol-induced NO formation is due to the redox-sensitive activation of the phosphatidylinositol 3-kinase/Akt pathway leading to direct eNOS activation subsequent to its phosphorylation on Ser 1177. Besides the phosphatidylinositol 3-kinase/Akt pathway, polyphenols have also been shown to activate eNOS by increasing the intracellular free calcium concentration and by activating estrogen receptors in ECs [45]. In addition to causing a rapid and sustained activation of eNOS by phosphorylation, polyphenols can increase the expression level of eNOS in ECs leading to an increased formation of NO [45]. Accordingly, genistein, at physiologically achievable concentrations in individuals consuming soy products, enhanced the expression of eNOS and subsequently elevated NO synthesis in both human aortic ECs (HAECs) and human umbilical vein ECs (HUVECs), with 1-10 μmol/L genistein inducing the maximal effects [46]. However, the effects of genistein on eNOS and NO were not mediated by activation of estrogen signaling or inhibition of tyrosine kinases, 2 known biological actions of genistein. Genistein (1–10 μmol/L) increased eNOS gene expression (1.8- to 2.6-fold of control) and significantly increased eNOS promoter activity of the human eNOS gene in HAEC and HUVEC, suggesting that genistein activates eNOS transcription [46]. Further, considering the effect of cyanidin-3-glucoside (Cy3G), a typical anthocyanin pigment, on eNOS expression, Xu *et al.* [47] observed that the treatment of bovine artery EC (BAECs) with Cy3G for 8 hours enhanced eNOS protein expression in a dose- and time-dependent manner. Longer incubation (12, 16, and 24 hours) of BAECs with 0.1 μmol/L of Cy3G caused a further increase in eNOS expression, and subsequently Cy3G also significantly increased NO output 2-fold (24 hours). Furthermore, Cy3G stimulated the phosphorylation of Src and extracellular signal-regulated kinase 1/2 (ERK1/2) in a time-dependent manner. Of interest, an Src kinase inhibitor, pp2, and MEK inhibitor, PD98059, blocked the ERK1/2 phosphorylation and eNOS expression. However, a recent meta-analysis by Hooper *et al.* [48] , aiming to systematically review the effectiveness of different flavonoid subclasses and flavonoid-rich food sources on cardiovascular disease and endothelial function, reported that soy protein isolate, isoflavone extracts, as well as red wine or grape and other flavanols do not present with significant effects on endothelial function. On the contrary, the data suggested a beneficial effect of black tea and chocolate or cocoa. Black tea increased FMD by 3.40% (95% confidential interval: 1.85%, 4.95%; one study), and chocolate or cocoa increased FMD by 1.45% (95% confidential interval: 0.62%, 2.28%; two studies), while flavonols caused a reduction in FMD (−1.4%; 95% confidential interval: −2.66, −0.14; one study). When data were available from ≥ 3 acute studies, only chocolate or cocoa significantly improved FMD (3.99%; 95% confidential interval: 2.86, 5.12; six studies). In addition, studies of red wine or grape and black tea suggested a modest benefit, although neither was statistically significant and both were significantly heterogeneous. For many of the subclasses, including anthocyanins, flavanones, green tea, and soyfoods, no published data on potential FMD effects were available. Only the group represented by chocolate or cocoa was able to show significant effects, both acutely and chronically, on FMD. Considering differences in chemical structures and range of doses, was observed significant heterogeneity between different flavonoid subgroups (*P *for heterogeneity < 0.01, *I*2= ≈ 80% in both acute and chronic studies). This confirmed that different flavonoid groups have different effects on FMD. Though not all the involved mechanisms have been exhaustively clarified, data from literature seem to suggest that flavonoids present with all the biological potential to positively affect vascular function via direct and indirect actions [49] (Figure 2). They potentially could be considered as healthy compounds for diet supplementation.

## 6. Conclusions

Cardiovascular risk factors contribute to oxidative stress, which causes an imbalance between NO and reactive oxygen species. This results in a relative decrease in NO bioavailability and/or in NO-soluble guanylate cyclase cascade in blood vessels. The disparity in protecting factors leads to endothelial and vascular smooth muscle cell dysfunction, resulting in increased tone and alterations in cell growth and gene expression that create a pro-thrombotic, pro-inflammatory environment [1,2,3,4]. This firstly triggers atherogenesis and then leads to formation, progression, and even destabilization of atherosclerotic plaques which, in turn, results in cardiovascular events and death [1,2,3,4]. Thus, NO clearly plays a pivotal role in the maintenance and repair of the vasculature, and a decrease in bioavailable NO is linked to adverse outcomes. This pathophysiological setting provides the rationale for exploring the potential therapeutic role for antioxidants and/or NO-donating agents in the prevention of cardiovascular disease. Nutrition plays an important role in the treatment of many diseases, and the right choice of nutrients can help to prevent disorders and improve the quality of life. Even in a `balanced' diet that meets macronutrient recommendations and micronutrient requirements, there is a growing body of evidence that bioactive compounds play an important role in optimizing health. Flavonoids, such as those occurring in tea and cocoa, are an example of a class of bioactive and antioxidant compounds that may confer beneficial effects on a number of important risk factors for cardiovascular disease. Nevertheless, protective antioxidant mechanisms are complex and multifactorial. Since the evidence of therapeutic effects of dietary polyphenols continues to accumulate, it is becoming of pivotal importance to understand the nature, the bioavailability and the putative pathophysiological mechanisms of action of this specific group of antioxidants. A better understanding of the mechanisms that underlie the biological effects of flavonoids could open the way for considering flavonoid supplementation and/or flavonoid-rich food ingestion as fundamental tools for human health deriving from daily diet. 

Currently, there is a scarcity of information concerning the amount of flavonoids that are needed on an acute or chronic basis to trigger the positive health effects discussed. Further, we should also clarify the potential bioactivity following synergistic and additive effects decreasing oxidative stress, improving endothelial dysfunction and thus protecting from atherosclerotic process. In addition, better understanding of the oxidative stress-dependent signal transduction mechanisms, their localization, and the integration of both reactive oxygen species-dependent transcriptional and signaling pathways in vascular pathophysiology is a prerequisite for effective pharmacological and non pharmacological interventions for cardiovascular protection from oxidative stress. However, additional studies should also clarify the role of flavonoids and polyphenol metabolites as bioactives, particularly focusing on specific target enzymes such as NADPH oxidases or lipoxygenases. This might be considered a new basis for molecular action of polyphenols [49].

Since their intake may reach 1 g/day, flavonoids represent an important source of antioxidants in daily diet. At the moment we could consider these antioxidant nutrients available in everyday life as a protective tool for prevention of atherosclerosis and cardiovascular disease. 
